# Why Do Big Data and Machine Learning Entail the Fractional Dynamics?

**DOI:** 10.3390/e23030297

**Published:** 2021-02-28

**Authors:** Haoyu Niu, YangQuan Chen, Bruce J. West

**Affiliations:** 1 Electrical Engineering and Computer Science Department, University of California, Merced, CA 95340, USA; hniu2@ucmerced.edu; 2 Mechanical Engineering Department, University of California, Merced, CA 95340, USA; 3 Office of the Director, Army Research Office, Research Triangle Park, NC 27709, USA; brucejwest213@gmail.com

**Keywords:** fractional calculus, fractional dynamics, fractional-order thinking, heavytailedness, big data, machine learning, variability, diversity

## Abstract

Fractional-order calculus is about the differentiation and integration of non-integer orders. Fractional calculus (FC) is based on fractional-order thinking (FOT) and has been shown to help us to understand complex systems better, improve the processing of complex signals, enhance the control of complex systems, increase the performance of optimization, and even extend the enabling of the potential for creativity. In this article, the authors discuss the fractional dynamics, FOT and rich fractional stochastic models. First, the use of fractional dynamics in big data analytics for quantifying big data variability stemming from the generation of complex systems is justified. Second, we show why fractional dynamics is needed in machine learning and optimal randomness when asking: “is there a more optimal way to optimize?”. Third, an optimal randomness case study for a stochastic configuration network (SCN) machine-learning method with heavy-tailed distributions is discussed. Finally, views on big data and (physics-informed) machine learning with fractional dynamics for future research are presented with concluding remarks.

## 1. Fractional Calculus (FC) and Fractional-Order Thinking (FOT)

Fractional calculus (FC) is the quantitative analysis of functions using non-integer-order integration and differentiation, where the order can be a real number, a complex number or even the function of a variable. The first recorded query regarding the meaning of a non-integer order differentiation appeared in a letter written in 1695 by Guillaume de l’Hôpital to Gottfried Wilhelm Leibniz, who at the same time as Isaac Newton, but independently of him, co-invented the infinitesimal calculus [1]. Numerous contributors have provided definitions for fractional derivatives and integrals [2] since then, and the theory along with the applications of FC have been expanded greatly over the centuries [3,4,5]. In more recent decades, the concept of **fractional dynamics** has merged and gained followers in the statistical and chemical physics communities [6,7,8]. For example, optimal image processing has improved through the use of fractional-order differentiation and fractional-order partial differential equations as summarized in Chen et al. [9,10,11]. Anomalous diffusion was described using fractional-diffusion equations in [12,13], and Metzler et al. used fractional Langevin equations to model viscoelastic materials [14].

Today, big data and machine learning (ML) are two of the hottest topics of applied scientific research, and they are closely related to one another. To better understand them, we also need fractional dynamics, as well as fractional-order thinking (FOT). Section 2 is devoted to the discussion of the relationships between big data, variability, and fractional dynamics, as well as to fractional-order data analytics (FODA) [15]. The topics touched on in this section include the Hurst parameter [16,17], fractional Gaussian noise (fGn), fractional Brownian motion (fBm), the fractional autoregressive integrated moving average (FARIMA) [18], the formalism of continuous time random walk (CTRW) [19], unmanned aerial vehicles (UAVs) and precision agriculture (PA) [20]. In Section 3, how to learn efficiently (optimally) for ML algorithms is investigated. The key to developing an efficient learning process is the method of optimization. Thus, it is important to design an efficient or perhaps optimal optimization method. The derivative-free methods, and the gradient-based methods, such as the Nesterov accelerated gradient descent (NAGD) [21], are both discussed. Furthermore, the authors propose designing and analyzing the ML algorithms in an S or Z transform domain in Section 3.3. FC is used in optimal randomness in the methods of stochastic gradient descent (SGD) [22] and random search, and in implementing the internal model principle (IMP) [23].

FOT is a way of thinking using FC. For example, there are non-integers between the integers; between logic 0 and logic 1, there is the fuzzy logic [24]; compared with integer-order splines, there are fractional-order splines [25]; between the high-order integer moments, there are non-integer-order moments, etc. FOT has been entailed by many research areas, for example, self-similar [26,27], scale-free or scale-invariant, power-law, long-range-dependence (LRD) [28,29], and $1/f^{\alpha }$ noise [30,31]. The terms porous media, particulate, granular, lossy, anomaly, disorder, soil, tissue, electrodes, biology [32], nano, network, transport, diffusion, and soft matters are also intimately related to FOT. However, in the present section, we mainly discuss **complexity and inverse power laws (IPL)**.

### 1.1. Complexity and Inverse Power Laws (IPLs)

**Figure 1:**
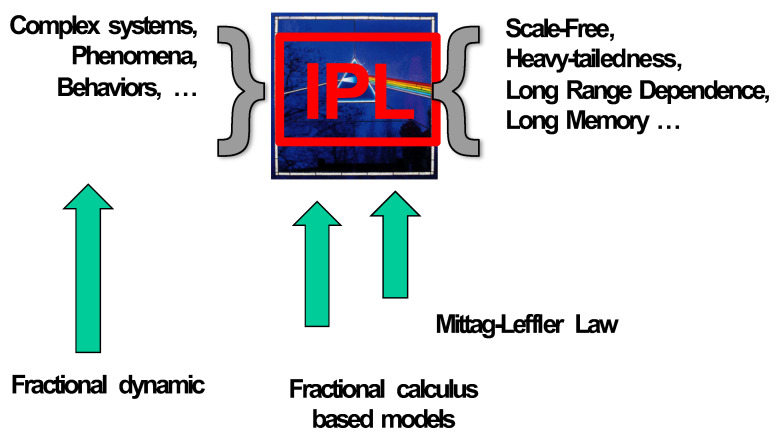
Inverse power law (complexity “bow tie”): On the left are the systems of interest that are thought to be complex. In the center panel, an aspect of the empirical data is characterized by an inverse power law (IPL). The right panel lists the potential properties associated with systems with data that have been processed and yield an IPL property. See text for more details.

When studying complexity, it is fair to ask, what does it mean to be complex? When do investigators begin identifying a system, network or phenomenon as complex [33,34]? There is an agreement among a significant fraction of the scientific community that when the distribution of the data associated with the process of interest obeys an IPL, the phenomenon is complex; see Figure 1. On the left side of the figure, the complexity “bow tie” [35,36,37,38] is the phenomenon of interest, thought to be a complex system. On the right side of the figure is the spectrum of system properties associated with IPL probability density functions (PDFs): the system has one or more of the properties of being scale-free, having a heavy tail, having a long-range dependence, and/or having a long memory [39,40]. In the book by West and Grigolini [41], there is a table listing a sample of the empirical power laws and IPLs uncovered in the past two centuries. For example, in scale-free networks, the degree distributions follow an IPL in connectivity [42,43]; in the processing of signals containing pink noise, the power spectrum follows an IPL [29]. For other examples, such as the probability density function (PDF), the autocorrelation function (ACF) [44], allometry ($Y=aX^{b}$) [45], anomalous relaxation (evolving over time) [46], anomalous diffusion (mean squared dissipation versus time) [13], and self-similarity can all be described by the IPL “bow tie” depicted in Figure 1.

The power law is usually described as:

(1) $$f\left(x\right)=ax^{k},$$

when *k* is negative, f(x) is an IPL. One important characteristic of this power law is scale invariance [47] determined by:

(2) $$f\left(cx\right)=a{\left(cx\right)}^{k}=c^{k}f\left(x\right)\propto f\left(x\right).$$

Note that when *x* is the time, the scaling depicts a property of the system dynamics. However, when the system is stochastic, the scaling is a property of the PDF (or correlation structure) and is a constraint on the collective properties of the system.

**Figure 2:**
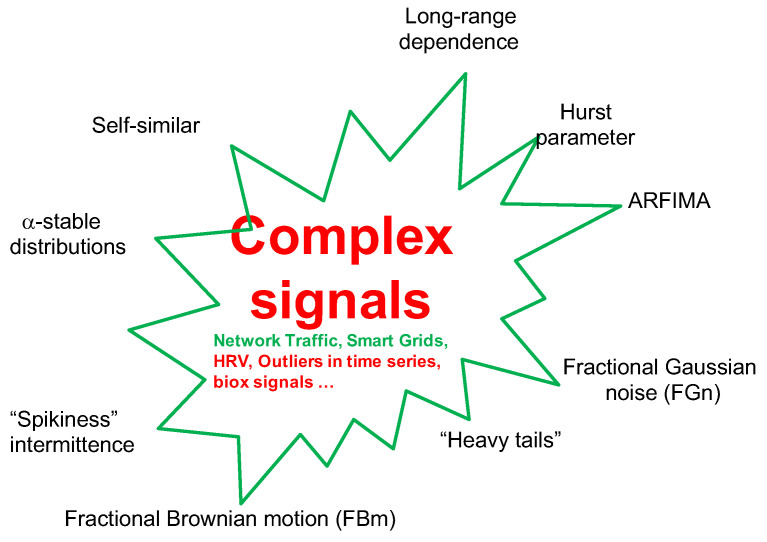
Complex signals (IPL): Here, the signal generated by a complex system is depicted. Exemplars of the systems are given as are the potential properties arising from the systems’ complexity.

FC is entailed by complexity, since an observable phenomenon represented by a fractal function has integer-order derivatives that diverge. Consequently, for the complexity characterization and regulation, we ought to use the fractional dynamics point of view because the fractional derivative of a fractal function is finite. Thus, complex phenomena, no matter whether they are natural or carefully engineered, ought to be described by fractional dynamics. Phenomena in complex systems in many cases should be analyzed using FC-based models, where mathematically, the IPL is actually the “Mittag–Leffler law” (MLL), which asymptotically becomes an IPL (Figure 2), known as heavy-tail behavior.

When an IPL results from processing data, one should think about how the phenomena can be connected to the FC. In [48], Gorenflo et al. explained the role of the FC in generating stable PDFs by generalizing the diffusion equation to one of fractional order. For the Cauchy problem, they considered the space-fractional diffusion equation:

(3) $$\frac{\partial u}{\partial t}=D\left(\alpha \right)\frac{\partial^{\alpha }u}{{\partial |x|}^{\alpha }},$$

where −∞<x<∞, t≥0 with u(x,0)=δ(x), 0<α≤2, and D(α) is a suitable diffusion coefficient. The fractional derivative in the diffusion variable is of the Reisz–Feller form, defined by its Fourier transform to be ${\left|k\right|}^{a}$. For the signalling problem, they considered the so-called time-fractional diffusion equation [49]:

(4) $$\frac{\partial^{2\beta }u}{\partial t^{2\beta }}=D\left(\beta \right)\frac{\partial^{2}u}{\partial x^{2}},$$

where x≥0, t≥0 with u(0,t)=δ(t), 0<β<1, and D(β) is a suitable diffusion coefficient. Equation (4) has also been investigated in [50,51,52]. Here, the Caputo fractional derivative in time is used.

There are rich forms in stochasticity [22], for example, heavytailedness, which corresponds to fractional-order master equations [53]. In Section 1.2, heavy-tailed distributions are discussed.

### 1.2. Heavy-Tailed Distributions

In probability theory, heavy-tailed distributions are PDFs whose tails do not decay exponentially [54]. Consequently, they have more weight in their tails than does an exponential distribution. In many applications, it is the right tail of the distribution that is of interest, but a distribution may have a heavy left tail, or both tails may be heavy. Heavy-tailed distributions are widely used for modeling in different disciplines, such as finance [55], insurance [56], and medicine [57]. The distribution of a real-valued random variable *X* is said to have a heavy right tail if the tail probabilities P(X>x) decay more slowly than those of any exponential distribution:

(5) $$\lim_{x\to \infty }\left(\frac{P(X>x)}{e^{-\lambda x}}\right)=\infty ,$$

for every λ>0 [58]. For the heavy left tail, an analogous definition can be constructed [59]. Typically, there are three important subclasses of heavy-tailed distributions: fat-tailed, long-tailed and subexponential distributions.

#### 1.2.1. Lévy Distribution

A Lévy distribution, named after the French mathematician Paul Lévy, can be generated by a random walk whose steps have a probability of having a length determined by a heavy-tailed distribution [60]. As a fractional-order stochastic process with heavy-tailed distributions, a Lévy distribution has better computational characteristics [61]. A Lévy distribution is stable and has a PDF that can be expressed analytically, although not always in closed form. The PDF of Lévy flight [62] is:

(6) $$p\left(x,\mu ,\gamma \right)=\left\{\begin{array}{cc} \frac{\sqrt{\frac{\gamma }{2\pi }}}{e^{\frac{\gamma }{2(x-\mu )}{\left(x-\mu \right)}^{3/2}}}, & x>\mu ,\\ 0, & x\leq \mu , \end{array}\right.$$

where μ is the location parameter and γ is the scale parameter. In practice, the Lévy distribution is updated by

(7) $$Lévy\left(\beta \right)=\frac{u}{{\left|\nu \right|}^{1/\beta }},$$

where *u* and ν are random numbers generated from a normal distribution with a mean of 0 and standard deviation of 1 [63]. The stability index β ranges from 0 to 2. Moreover, it is interesting to point out that the well-known Gaussian and Cauchy distributions are special cases of the Lévy PDF when the stability index is set to 2 and 1, respectively.

#### 1.2.2. Mittag–Leffler PDF

The Mittag–Leffler PDF [64] for the time interval between events can be written as a mixture of exponentials with a known PDF for the exponential rates:

(8) $$E_{\theta }\left(-t^{\theta }\right)=\int_{0}^{\infty }exp\left(-\mu t\right)g\left(\mu \right)d\mu ,$$

with a weight for the rates given by:

(9) $$g\left(\mu \right)=\frac{1}{\pi }\frac{\sin(\theta \pi )}{\mu^{1+\theta }+2\cos\left(\theta \pi \right)\mu +\mu^{1-\theta }}.$$

The most convenient expression for the random time interval was proposed by [65]:

(10) $$\tau_{\theta }=-\gamma_{t}{\left(\ln u\frac{\sin(\theta \pi )}{\tan(\theta \pi v)}-\cos\left(\theta \pi \right)\right)}^{1/\theta },$$

where *u*, *v*∈ (0,1) are independent uniform random numbers, $\gamma_{t}$ is the scale parameter, and $\tau_{\theta }$ is the Mittag–Leffler random number. In [66], Wei et al. used the Mittag–Leffer distribution for improving the Cuckoo Search algorithm, which did show an improved performance.

#### 1.2.3. Weibull Distribution

A random variable is described by a Weibull distribution if it has a PDF function *F*:

(11) $$F\left(x\right)=e^{-{\left(x/k\right)}^{\lambda_{w}}},$$

where k>0 is the scale parameter, and $\lambda_{w}>0$ is the shape parameter [67]. If the shape parameter is $\lambda_{w}<1$, the Weibull distribution is determined to be heavy tailed.

#### 1.2.4. Cauchy Distribution

A random variable is described by a Cauchy PDF if its cumulative distribution is [68,69]:

(12) $$F\left(x\right)=\frac{1}{\pi }\arctan\left(\frac{2(x-\mu_{c})}{\sigma }\right)+\frac{1}{2},$$

where $\mu_{c}$ is the location parameter and σ is the scale parameter. Cauchy distributions are examples of fat-tailed distributions, which have been empirically encountered in a variety of areas including physics, earth sciences, economics and political science [70]. Fat-tailed distributions include those whose tails decay like an IPL, which is a common point of reference in their use in the scientific literature [71]:

#### 1.2.5. Pareto Distribution

**Figure 3:**
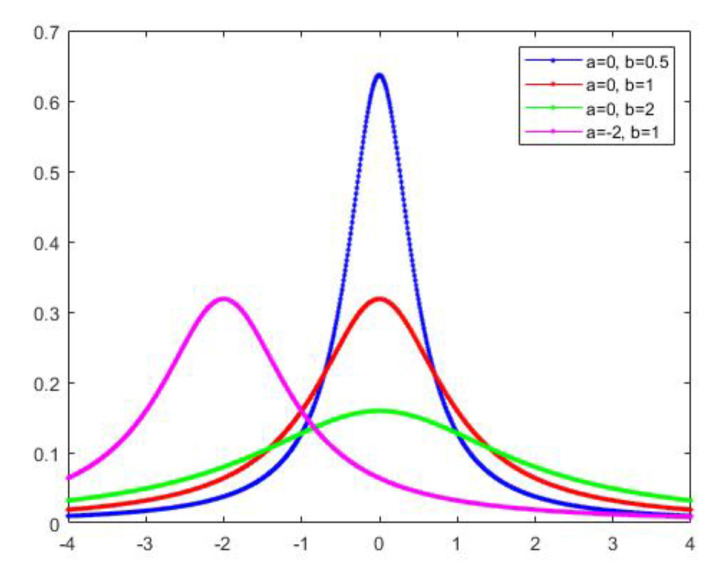
Cauchy distributions are examples of fat-tailed distributions. The parameter a is the location parameter; the parameter b is the scale parameter.

A random variable is said to be described by a Pareto PDF if its cumulative distribution function is

(13) $$F\left(x\right)=\left\{\begin{array}{c} 1-{\left(\frac{b}{x}\right)}^{a},x\geq b,\\ 0,x<b, \end{array}\right.$$

where b>0 is the scale parameter and a>0 is the shape parameter (Pareto’s index of inequality) [72] (Figure 3).

#### 1.2.6. The α-Stable Distribution

**Figure 4:**
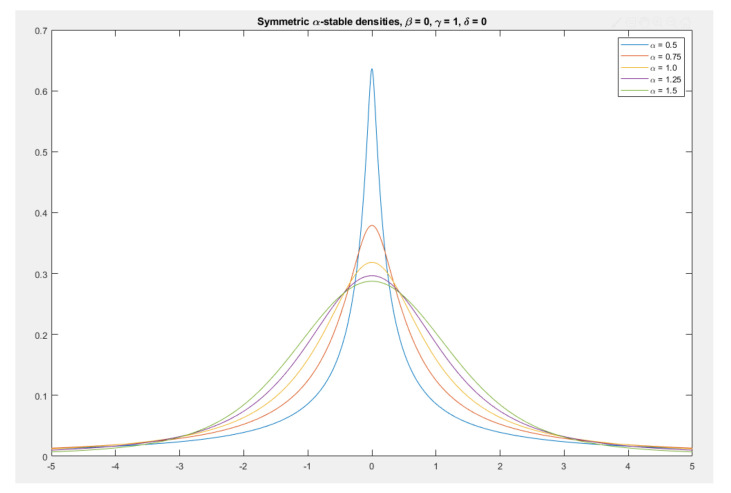
Symmetric α-stable distributions with unit scale factor. The most narrow probability density function (PDF) shown has the smallest IPL index and, consequently, the most weight in the tail regions.

A PDF is said to be stable if a linear combination of two independent random variables, each with the same distribution, has the same distribution for the conjoined variable. This PDF is also called the Lévy α-stable distribution [73,74]. Since the normal distribution, Cauchy distribution and Lévy distribution all have the above property, one can consider them to be special cases of stable distributions. Stable distributions have 0 < α≤ 2, with the upper bound corresponding to the normal distribution, and α = 1, to the Cauchy distribution (Figure 4). The PDFs have undefined variances for α < 2, and undefined means for α≤ 1. Although their PDFs do not admit a closed-form formula in general, except in special cases, they decay with an IPL tail called the IPL index, which determines the behavior of the PDF. As the IPL index gets smaller, the PDF acquires a heavier tail. An example of an IPL index analysis is given in Section 1.4.

### 1.3. Mixture Distributions

A mixture distribution is derived from a collection of other random variables. First, a random variable is selected by chance from the collection according to given probabilities of selection. Then, the value of the selected random variable is realized. The mixture PDFs are complicated in terms of simpler PDFs, which provide a good model for certain datasets. The different subsets of the data can exhibit different characteristics. Therefore, the mixed PDFs can effectively characterize the complex PDFs of certain real-world datasets. In [75], a robust stochastic configuration network (SCN) based on a mixture of Gaussian and Laplace PDFs was proposed. Thus, Gaussian and Laplace distributions are mentioned in this section for comparison purposes.

#### 1.3.1. Gaussian Distribution

A random variable *X* has a Gaussian distribution with the mean $\mu_{G}$ and variance $\sigma_{G}^{2}$ (−∞ < $\mu_{G}$ < *∞* and $\sigma_{G}$ > 0) if *X* has a continuous distribution for which the PDF is as follows [76]:

(14) $$f\left(x|\mu_{G},\sigma_{G}^{2}\right)=\frac{1}{{\left(2\pi \right)}^{1/2}\sigma_{G}}e^{-\frac{1}{2}{\left(\frac{x-\mu_{G}}{\sigma_{G}}\right)}^{2}},for\;-\infty <x<\infty .$$

#### 1.3.2. Laplace Distribution

The PDF of the Laplace distribution can be written as follows [75]:

(15) $$F\left(x|\mu_{l},\eta \right)=\frac{1}{{\left(2\eta^{2}\right)}^{1/2}}e^{\left(-\frac{\sqrt{2}\left|x-\mu_{l}\right|}{\eta }\right)},$$

where $\mu_{l}$ and η represent the location and scale parameters, respectively.

### 1.4. IPL Tail-Index Analysis

There are two approaches to the problem of the IPL tail-index estimation: the parametric [77] and the nonparametric [78]. To estimate the tail index using the parametric approach, some researchers employ generalized a extreme value (GEV) distribution [79] or Pareto distribution, and they may apply the maximum-likelihood estimator (MLE).

The stochastic gradient descent (SGD) has been widely used in deep learning with great success because of the computational efficiency [80,81]. The gradient noise (GN) in the SGD algorithm is often considered to be Gaussian in the large data regime by assuming that the classical central limit theorem (CLT) kicks in. The machine-learning tasks are usually considered as solving the following optimization problem:

(16) $$w^{*}=argmin\left\{f\left(w\right)≜\frac{1}{n}\sum_{i=1}^{n}f^{\left(i\right)}\left(w\right)\right\},$$

where *w* denotes the weights of the neural network, *f* denotes the loss function, and *n* denotes the total number of instances. Then, the SGD is calculated based on the following iterative scheme:

(17) $$w_{k+1}=w_{k}-\eta \nabla f_{k}\left(w_{k}\right),$$

where *k* means the iteration number, and $\nabla f_{k}\left(w_{k}\right)$ denotes the stochastic gradient at iteration *k*.

**Figure 5:**
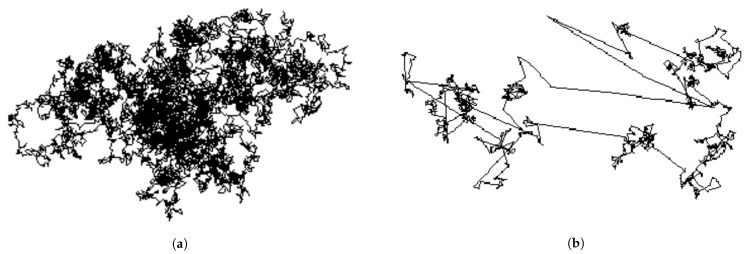
(**a**) Brownian motion; (**b**) Lévy motion. Note that both figures are at the same size scale.

**Figure 6:**
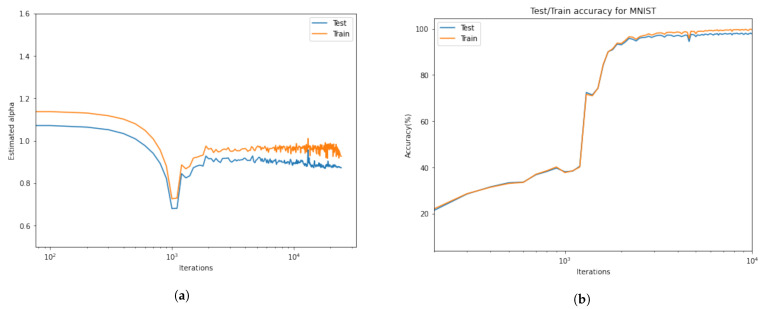
(**a**) The behavior of tail-index α during the iterations; (**b**) The training and testing accuracy. At first, the α decreases very slowly; when α reaches its lowest level, which means longer tail distribution, there is a significant jump, which causes a sudden decrease in accuracy. Beyond this point, the process recovers again, and we see stationary behavior in α and an increasing behavior in the accuracy.

Since the gradient noise might not be Gaussian, the use of Brownian motion would not be appropriate to represent its behavior. Therefore, Şimşekli et al. replaced the gradient noise with the α-stable Lévy motion [82], whose increments have an α-stable distribution [83]. Because of the heavy-tailed nature of the α-stable distribution, the Lévy motion might incur large, discontinuous jumps [84], and therefore, it would exhibit a fundamentally different behavior than would Brownian motion (Figure 5):

Figure 6 shows that there are two distinct phases of SGD (in this configuration, before and after iteration 1000). At first, the loss decreases very slowly, the accuracy slightly increases, and more interestingly, α rapidly decreases. When α reaches its lowest level, which means a longer tail distribution, there is a significant jump, which causes a sudden decrease in accuracy. Beyond this point, the process recovers again, and we see stationary behavior in α and an increasing behavior in the accuracy.

## 2. Big Data, Variability and FC

The term “big data” started showing up in the early 1990s. The world’s technological per capita capacity to store information has roughly doubled every 40 months since the 1980s [85]. Since 2012, there have been 2.5 exabytes (2.5 × 2${}^{60}$ bytes) of data generated every day [86]. According to data report predictions, there will be 163 zettabytes of data by 2025 [87]. Firican proposed, in [88], ten characteristics (properties) of big data to prepare for both the challenges and advantages of big data initiatives (Table 1). In this article, **variability** is the most important characteristic being discussed. Variability refers to several properties of big data. First, it refers to the number of inconsistencies in the data, which need to be understood by using anomaly- and outlier-detection methods for any meaningful analytics to be performed. Second, variability can also refer to diversity [89,90], resulting from disparate data types and sources, for example, healthy or unhealthy [91,92]. Finally, variability can refer to multiple research topics (Table 2).

Considering variability, Xunzi (312 BC–230 BC), who was a Confucian philosopher, made a useful observation: “Throughout a thousand acts and ten thousand changes, his way remains one and the same” [93]. Therefore, we ask: what is the “one and the same” for big data? This is the **variability**, which refers to the behavior of the dynamic system. The ancient Greek philosopher Heraclitus (535 BC–475 BC) also realized the importance of variability, prompting him to say: “The only thing that is constant is change”; “It is in changing that we find purpose”; “Nothing endures but change”; “No man ever steps in the same river twice, for it is not the same river and he is not the same man”.

Heraclitus actually recognized the (fractional-order) dynamics of the river without modern scientific knowledge (in nature). After two thousand years, the integer-order calculus was invented by Sir Issac Newton and Gottfried Wilhelm Leibniz, whose main purpose was to quantify that change [94,95]. From then, scientists started using integer-order calculus to depict dynamic systems, differential equations, modelling, etc. In the 1950s, Scott Blair, who first introduced the FC into rheology, pointed out that the integer-order dynamic view of change is only for our own “convenience” (a little bit selfish). In other words, denying fractional calculus is equivalent to denying the existence of non-integers between the integers!

Blair said [96]: “We may express our concepts in Newtonian terms if we find this convenient but, if we do so, we must realize that we have made a translation into a language which is foreign to the system which we are studying (1950)”.

Therefore, variability exists in big data. However, how do we realize the modeling, analysis and design (MAD) for the variability in big data within complex systems? We need fractional calculus! In other words, big data are at the nexus of complexity and FC. Thus, we first proposed fractional-order data analytics (FODA) in 2015. Metrics based on using the fractional-order signal processing techniques should be used for quantifying the generating dynamics of observed or perceived variability [15].

### 2.1. Hurst Parameter, fGn, and fBm

The Hurst parameter or Hurst exponent (*H*) was proposed for the analysis of the long-term memory of time series. It was originally developed to quantify the long-term storage capacity of reservoirs for the Nile river’s volatile rain and drought conditions more than a half century ago [16,17]. To date, the Hurst parameter has also been used to measure the intensity of long range dependence (LRD) in time series [97], which requires accurate modeling and forecasting. The self-similarity and the estimation of the statistical parameters of LRD have commonly been investigated recently [98]. The Hurst parameter has also been used for characterizing the LRD process [97,99]. A LRD time series is defined as a stationary process that has long-range correlations if its covariance function C(n) decays slowly as:

(18) $$\lim_{n\to \infty }\frac{C(n)}{n^{-\alpha }}=c,$$

where 0<α<1, which relates to the Hurst parameter according to α=2−2H[100,101]. The parameter *c* is a finite, positive constant. When the value of *n* is large, C(n) behaves as the IPL $c/n^{\alpha }$[102]. Another definition for an LRD process is that the weakly stationary time-series X(t) is said to be LRD if its power spectral density (PSD) follows:

(19) $$f\left(\lambda \right)∼C_{f}{\left|\lambda \right|}^{-\beta },$$

as λ→ 0, for a given $C_{f}>0$ and a given real parameter β∈ (0,1), which corresponds to H=(1+β)/2 [103]. When 0 < *H* < 0.5, it indicates that the time intervals constitute a negatively correlated process. When 0.5 < *H* < 1, it indicates that time intervals constitute a positively correlated process. When H=0.5, it indicates that the process is uncorrelated.

Two of the most common LRD processes are fBm [104] and fGn [105]. The fBm process with H(0<H<1) is defined as:

(20) $$B_{H}\left(t\right)=\frac{1}{\Gamma (H+1/2)}\left\{\int_{-\infty }^{0}\left[{\left(t-s\right)}^{H-1/2}-{\left(-s\right)}^{H-1/2}\right]dW\left(s\right)+\int_{0}^{t}{\left(t-s\right)}^{H-1/2}dW\left(s\right)\right\},$$

where *W* denotes a Wiener process defined on (−∞,∞) [106]. The fGn process is the increment sequences of the fBm process, defined as:

(21) $$X_{k}=Y\left(k+1\right)-Y\left(k\right),$$

where Y(k) is a fBm process [107].

### 2.2. Fractional Lower-Order Moments (FLOMs)

The FLOM is based on α-stable PDFs. The PDFs of an α-stable distribution decay in the tails more slowly than a Gaussian PDF does. Therefore, for sharp spikes or occasional bursts in signals, an α-stable PDF can be used for characterizing signals more frequently than Gauss-distributed signals [108]. Thus, the FLOM plays an important role in impulsive processes [109], equivalent to the role played by the mean and variance in a Gaussian processes. When 0 <α≤ 1, the α-stable processes have no finite first- or higher-order moments; when 1 < α < 2, the α-stable processes have a first-order moment and all the FLOMs with moments of fractional order that is less than 1. The correlation between the FC and FLOM was investigated in [110,111]. For the Fourier-transform pair p(x) and ϕ(μ), the latter is the characteristic function and is the Fourier transform of the PDF; a complex FLOM can have complex fractional lower orders [110,111]. A FLOM-based fractional power spectrum includes a covariation spectrum and a fractional low-order covariance spectrum [112]. FLOM-based fractional power spectrum techniques have been successfully used in time-delay estimation [112].

### 2.3. Fractional Autoregressive Integrated Moving Average (FARIMA) and Gegenbauer Autoregressive Moving Average (GARMA)

A continuous-time linear time-invariant (LTI) system can be characterized using a linear difference equation, which is known as an autoregression and moving average (ARMA) model [113,114]. The process $X_{t}$ of ARMA(p,q) is defined as:

(22) $$\Phi \left(B\right)X_{t}=\Theta \left(B\right)\epsilon_{t},$$

where $\epsilon_{t}$ is white Gaussian noise (wGn), and *B* is the backshift operator. However, the ARMA model can only describe a short-range dependence (SRD) property. Therefore, based on the Hurst parameter analysis, more suitable models, such as FARIMA [115,116] and fractional integral generalized autoregressive conditional heteroscedasticity (FIGARCH) [117], were designed to more accurately analyze the LRD processes. The most important feature of these models is the long memory characteristic. The FARIMA and FIGARCH can capture both the short- and the long-memory nature of time series. For example, the FARIMA process $X_{t}$ is usually defined as [118]:

(23) $$\Phi \left(B\right){\left(1-B\right)}^{d}X_{t}=\Theta \left(B\right)\epsilon_{t},$$

where d∈(−0.5,0.5), and ${\left(1-B\right)}^{d}$ is a fractional-order difference operator. The locally stationary long-memory FARIMA model has the same equation as that of Equation (23), except that *d* becomes $d_{t}$, which is a time-varying parameter [119]. The locally stationary long-memory FARIMA model captures the local self-similarity of the system.

The generalized locally stationary long-memory process FARIMA model was investigated in [119]. For example, a generalized FARIMA model, which is called the Gegenbauer autoregressive moving average (GARMA), was introduced in [120]. The GARMA model is defined as:

(24) $$\Phi \left(B\right){\left(1-2uB+B^{2}\right)}^{d}X_{t}=\Theta \left(B\right)\epsilon_{t},$$

where u∈[−1,1], which is a parameter that can control the frequency at which the long memory occurs. The parameter *d* controls the rate of decay of the autocovariance function. The GARMA model can also be extended to the so-called “*k*-factor GARMA model”, which allows for long-memory behaviors to be associated with each of *k* frequencies (Gegenbauer frequencies) in the interval [0, 0.5] [121].

### 2.4. Continuous Time Random Walk (CTRW)

The CTRW model was proposed by Montroll and Weiss as a generalization of diffusion processes to describe the phenomenon of anomalous diffusion [19]. The basic idea is to calculate the PDF for the diffusion process by replacing the discrete steps with continuous time, along with a PDF for step lengths and a waiting-time PDF for the time intervals between steps. Montroll and Weiss applied random intervals between the successive steps in the walking process to account for local structure in the environment, such as traps [122]. The CTRW has been used for modeling multiple complex phenomena, such as chaotic dynamic networks [123]. The correlation between CTRW and diffusion equations with fractional time derivatives has also been established [124]. Meanwhile, time-space fractional diffusion equations can be treated as CTRWs with continuously distributed jumps or continuum approximations of CTRWs on lattices [125].

### 2.5. Unmanned Aerial Vehicles (UAVs) and Precision Agriculture

**Figure 7:**
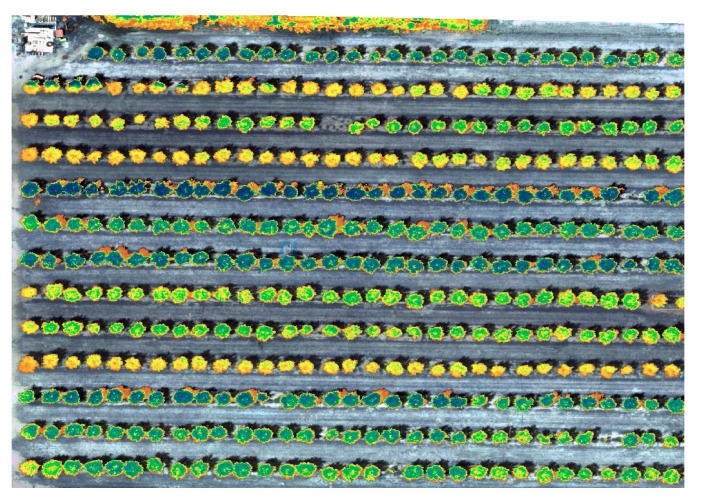
Normalized difference vegetation index (NDVI) mapping of pomegranate trees.

As a new remote-sensing platform, researchers are more and more interested in the potential of small UAVs for precision agriculture [126,127,128,129,130,131,132,133,134,135,136], especially for heterogeneous crops, such as vineyards and orchards [137,138]. Mounted on UAVs, lightweight sensors, such as RGB cameras, multispectral cameras and thermal infrared cameras, can be used to collect high-resolution images. The higher temporal and spatial resolutions of the images, relatively low operational costs, and nearly real-time image acquisition make the UAVs an ideal platform for mapping and monitoring the variability of crops and trees. UAVs can create big data and demand the FODA due to the “complexity” and, thus, variability inherent in the life process. For example, Figure 7 shows the normalized difference vegetation index (NDVI) mapping of a pomegranate orchard at a USDA ARS experimental field. Under different irrigation levels, the individual trees can show strong variability during the analysis of water stress. Life is complex! Thus, it entails variability, which as discussed above, in turn, entails fractional calculus. UAVs can then become “Tractor 2.0” for farmers in precision agriculture.

## 3. Optimal Machine Learning and Optimal Randomness

**Machine learning (ML)** is the science (and art) of programming computers so they can learn from data [139]. A more engineering-oriented definition was given by Tom Mitchell in 1997 [140], “A computer program is said to learn from experience E with respect to some task T and some performance measure P, if its performance on T, as measured by P, improves with experience E”.

Most ML algorithms perform training by solving optimization problems that rely on first-order derivatives (Jacobians), which decide whether to increase or decrease weights. For huge speed boosts, faster optimizers are being used instead of the regular gradient descent optimizer. For example, the most popular boosters are momentum optimization [141], Nesterov acelerated gradient [21], AdaGrad [142], RMSProp [143] and Adam optimization [144]. The second-order (Hessian) optimization methods usually find the solutions with faster rates of convergence but with higher computational costs. Therefore, the answer to the following question is important: what is a more optimal ML algorithm? What if the derivative is fractional order instead of integer order? In this section, we discuss some applications of fractional-order gradients to optimization methods in machine-learning algorithms and investigate the accuracy and convergence rates.

**Figure 8:**
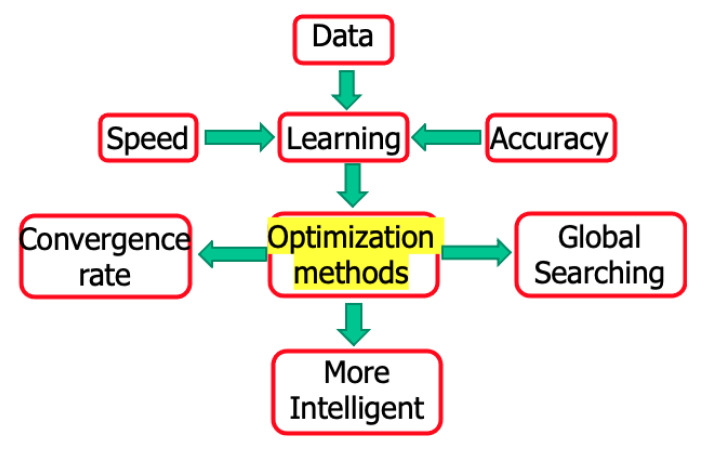
Data analysis in nature.

As mentioned in the big data section, there is a huge amount of data in human society and nature. During the learning process of ML, we care not only about the speed, but also the accuracy of the data the machine is learning (Figure 8). The learning algorithm is important; otherwise, the data labeling and other labor costs will exhaust people beyond their abilities. When applying the accoladed artificial intelligence (AI) to an algorithm, a strong emphasis is on artificial, only followed weakly by intelligence. Therefore, the key to ML is what optimization methods are being applied. The convergence rate and global searching are two important parts of the optimization method.

**Reflection:** ML is, today, a hot research topic and will probably remain so into the near future. How a machine can learn efficiently (optimally) is always important. The key for the learning process is the optimization method. Thus, in designing an efficient optimization method, it is necessary to answer the following three questions:

- What is the optimal way to optimize?
- What is the **more optimal** way to optimize?
- Can we demand **“more optimal machine learning”**, for example, deep learning with the minimum/smallest labeled data)?

**Optimal randomness:** In the section on the Lévy PDF, the Lévy flight is the search strategy for food the albatross has developed over millions of years of evolution. Admittedly, this is a slow optimization procedure [84]. From this perspective, we should call “Lévy distribution” an optimized or learned randomness used by albatrosses for searching for food. Therefore, we pose the question: “can the search strategy be more optimal than Lévy flight?” The answer is yes if one adopts the FC [145]! Optimization is a very complex area of study. However, a few studies have investigated using FC to obtain a better optimization strategy.

Theoretically, there are two broad optimization categories; these are derivative-free and gradient-based. For the derivative-free methods, there are the direct-search methods, consisting of particle swarm optimization (PSO) [146,147], etc. For the gradient-based methods, there are gradient descent and its variants. Both of the two categories have shown better performance when using the FC as demonstrated below.

### 3.1. Derivative-Free Methods

**Figure 9:**
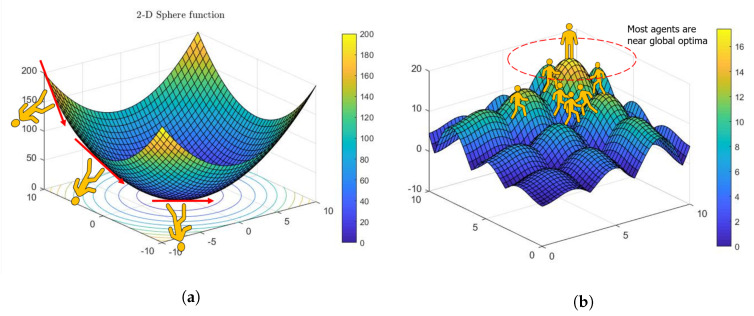
The 2-D Alpine function for derivative-free methods; there are (**a**) single agent search and (**b**) swarm-based search methods.

**Figure 10:**
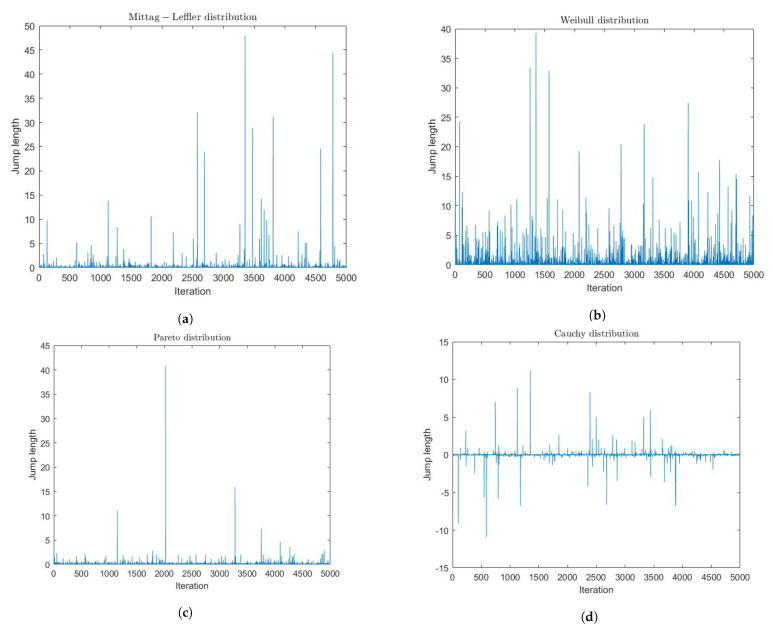
Sample paths. Wei et al. [148] investigated the optimal randomness in a swarm-based search. Four heavy-tailed PDFs were used for sample path analysis; there are (**a**) Mittag-Leffler distribution, (**b**) Weibull distribution, (**c**) Pareto distribution, and (**d**) Cauchy distribution. The Long steps, referring to the jump length, frequently happened for all distributions, which showed strong heavy-tailed performance. For more details, please refer to [148].

For derivative-free methods, there are single agent search and swarm-based search methods (Figure 9). Exploration is often achieved by randomness or random numbers in terms of some predefined PDFs. Exploitation uses local information such as gradients to search local regions more intensively, and such intensification can enhance the rate of convergence. Thus, a question was posed: what is the optimal randomness? Wei et al. [148] investigated the optimal randomness in a swarm-based search. Four heavy-tailed PDFs have been used for sample path analysis (Figure 10). Based on the experimental results, the randomness-enhanced cuckoo search (CS) algorithms [66,149,150] can identify the unknown specific parameters of a fractional-order system with better effectiveness and robustness. The randomness-enhanced CS algorithms can be considered as a promising tool for solving real-world complex optimization problems. The reason is that optimal randomness is applied with fractional-order noise during the exploration, which is more optimal than the “optimized PSO”, CS. The fractional-order noise refers to the stable PDFs [48]. In other words, when we are discussing optimal randomness, we are discussing fractional calculus!

### 3.2. The Gradient-Based Methods

The gradient descent (GD) is a very common optimization algorithm, which can find the optimal solutions by iteratively tweaking parameters to minimize the cost function. The stochastic gradient descent (SGD) randomly selects times during the training process. Therefore, the cost function bounces up and down, decreasing on average, which is good for escape from local optima. Sometimes, noise is added into the GD method, and usually, such noise follows a Gaussian PDF in the literature. We ask, “why not heavy-tailed PDFs”? The answer to this question could lead to interesting future research.

#### Nesterov Accelerated Gradient Descent (NAGD)

**Figure 11:**
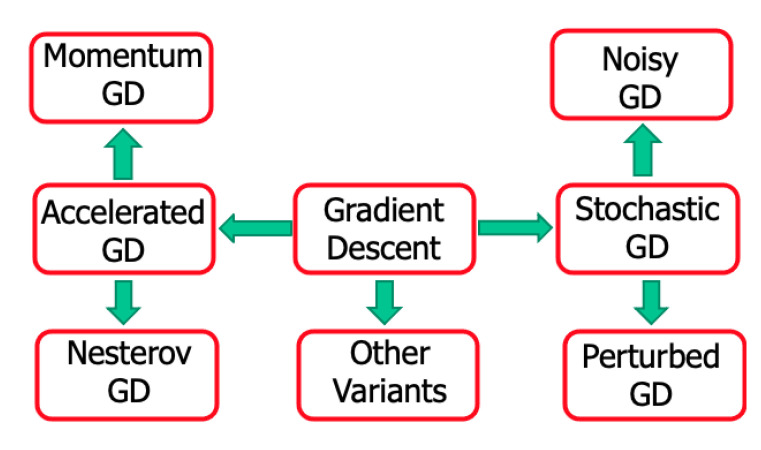
Gradient descent and its variants.

There are many variants of GD analysis as suggested in Figure 11. One of the most popular methods is the NAGD [21]:

(25) $$\left\{\begin{array}{c} y_{k+1}=ay_{k}-\mu \nabla f\left(x_{k}\right),\\ x_{k+1}=x_{k}+y_{k+1}+by_{k}, \end{array}\right.$$

where by setting b=−a/(1+a), one can derive the NAGD. When b=0, one can derive the momentum GD. The NAGD can also be formulated as:

(26) $$\left\{\begin{array}{c} x_{k}=y_{k-1}-\mu \nabla f\left(y_{k-1}\right),\\ y_{k}=x_{k}+\frac{k-1}{k+2}\left(x_{k}-x_{k-1}\right). \end{array}\right.$$

Set $t=k\sqrt{\mu }$, and one can, in the continuous limit, derive the corresponding differential equation:

(27) $$\ddot{X}+\frac{3}{t}\dot{X}+\nabla f\left(X\right)=0.$$

The main idea of Jordan’s work [151] is to analyze the iteration algorithm in the continuous-time domain. For differential equations, one can use the Laypunov or variational method to analyze the properties; for example, the convergence rate is $O(\frac{1}{t^{2}})$. One can also use the variational method to derive the master differential equation for an optimization method, such as the least action principle [152], Hamilton’s variational principle [153] and the quantum-mechanical path integral approach [154]. Wilson et al. [151] built a Euler–Lagrange function to derive the following equation:

(28) $${\ddot{X}}_{t}+2\gamma {\dot{X}}_{t}+\frac{\gamma^{2}}{\mu }\nabla f\left(X_{t}\right)=0.$$

which is in the same form as the master differential equation of NAGD.

Jordan’s work revealed that one can transform an iterative (optimization) algorithm to its continuous-time limit case, which can simplify the analysis (Laypunov methods). One can directly design a differential equation of motion (EOM) and then discretize it to derive an iterative algorithm (variational method). The key is to find a suitable Laypunov functional to analyze the stability and convergent rate. The new exciting fact established by Jordan is that optimization algorithms can be systematically synthesized using Lagrangian mechanics (Euler–Lagrange) through EOMs.

Thus, is there an optimal way to optimize using optimization algorithms stemming from Equation (28)? Obviously, why not an equation such as Equation (28) of fractional order? Considering the ${\dot{X}}_{t}$ as $X_{t}^{\left(\alpha \right)}$, it will provide us with more research possibilities, such as the fractional-order calculus of variation (FOCV) and fractional-order Euler–Lagrange (FOEL) equation. For the SGD, optimal randomness using the fractional-order noises can also offer better than the best performance, similarly shown by Wei et al. [148].

### 3.3. What Can the Control Community Offer to ML?

In the IFAC 2020 World Congress Pre-conference Workshop, Eric Kerrigan proposed “The Three Musketeers” that the control community can contribute to ML [155]. These three are the IMP [23], the Nu-Gap metric [156] and model discrimination [157]. Herein, we focused on the IMP. Kashima et al. [158] transferred the convergence problem of numerical algorithms into a stability problem of a discrete-time system. An et al. [159] explained that the commonly used SGD-momentum algorithm in ML is a PI controller and designed a PID algorithm. Motivated by [159] but differently from M. Jordan’s work, we proposed designing and analyzing the algorithms in the *S* or *Z* domain. Remember that GD is a first-order algorithm:

(29) $$x_{k+1}=x_{k}-\mu \nabla f\left(x_{k}\right),$$

where μ>0 is the step size (or learning rate). Using the Z transform, one can achieve:

(30) $$X\left(z\right)=\frac{\mu }{z-1}{\left[-\nabla f\left(x_{k}\right)\right]}_{z}.$$

Approximate the gradient around the extreme point $x^{*}$, and one can obtain:

(31) $$\nabla f\left(x_{k}\right)\approx A\left(x_{k}-x^{*}\right),\;with\;A=\nabla^{2}f\left(x^{*}\right).$$

**Figure 12:**
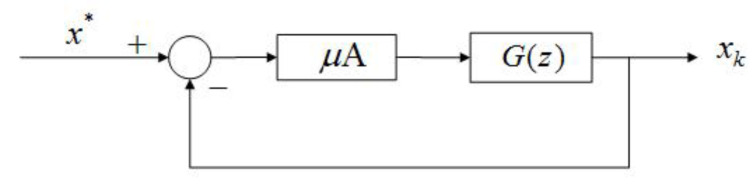
The integrator model (embedded in G(z)). The integrator in the forward loop eliminates the tracking steady-state error for a constant reference signal (internal model principle (IMP)).

For the plain GD in Figure 12, we have G(z)=1/(z−1), which is an integrator. For fractional-order GD (FOGD), the updating term of $x_{k}$ in Equation (29) can be treated as a filtered gradient signal. In [160], Fan et al. shared similar thoughts: “Accelerating the convergence of the moment method for the Boltzmann equation using filters”. The integrator in the forward loop eliminates the tracking error for a constant reference signal according to the internal model principle (IMP). Similarly, the GD momentum (GDM) designed to accelerate the conventional GD, which is popularly used in ML, can be analyzed using Figure 12 by:

(32) $$\left\{\begin{array}{c} y_{k+1}=\alpha y_{k}-\mu \nabla f\left(x_{k}\right),\\ x_{k+1}=x_{k}+y_{k+1}, \end{array}\right.$$

where $y_{k}$ is the accumulation of the history gradient and α∈(0,1) is the rate of the moving average decay. Using the Z transform for the update rule, one can derive:

(33) $$\left\{\begin{array}{c} zY\left(z\right)=\alpha Y\left(z\right)-\mu {\left[\nabla f\left(x_{k}\right)\right]}_{z},\\ zX(z)=X(z)+zY(z). \end{array}\right.$$

Then, after some algebra, one obtains the following equation:

(34) $$X\left(z\right)=\frac{\mu z}{\left(z-1)(z-\alpha \right)}{\left[-\nabla f\left(x_{k}\right)\right]}_{z}.$$

For the GD momentum, we have $G\left(z\right)=\frac{z}{\left(z-1)(z-\alpha \right)}$ in Figure 12, with an integrator in the forward loop. The GD momentum is a second-order (G(z)) algorithm with an additional pole at z=α and one zero at z=0. The “second-order” refers to the order of G(z), which makes it different from the algorithm using the Hessian matrix information. Moreover, NAGD can be simplified as:

(35) $$\left\{\begin{array}{c} y_{k+1}=x_{k}-\mu \nabla f\left(x_{k}\right),\\ x_{k+1}=\left(1-\lambda \right)y_{k+1}+\lambda y_{k}, \end{array}\right.$$

where μ is the step size and λ is a weighting coefficient. Using the Z transform for the update rule, one can derive:

(36) $$\left\{\begin{array}{c} zY\left(z\right)=X\left(z\right)-\mu {\left[\nabla f\left(x_{k}\right)\right]}_{z},\\ zX(z)=(1-\lambda )zY(z)+\lambda Y(z). \end{array}\right.$$

Different from the GD momentum, and after some algebra, one obtains:

(37) $$X\left(z\right)=\frac{-(1-\lambda )z-\lambda }{\left(z-1)(z+\lambda \right)}\mu {\left[\nabla f\left(x_{k}\right)\right]}_{z}=\frac{z+\frac{\lambda }{1-\lambda }}{\left(z-1)(z+\lambda \right)}\mu \left(1-\lambda \right){\left[-\nabla f\left(x_{k}\right)\right]}_{z}.$$

For NAGD, we have $G\left(z\right)=\frac{z+\frac{\lambda }{1-\lambda }}{\left(z-1)(z+\lambda \right)}$, again, with an integrator in the forward loop (Figure 12). NAGD is a second-order algorithm with an additional pole at z=−λ and a zero at $z=\frac{-\lambda }{1-\lambda }$.

“Can G(z) be of higher order or fractional order”? Of course it can! As shown in Figure 12, a necessary condition for the stability of an algorithm is that all the poles of the closed-loop system are within the unit disc. If the Lipschitz continuous gradient constant *L* is given, one can replace *A* with *L*, and then, the condition is sufficient. For each G(z), there is a corresponding iterative optimization algorithm. G(z) can be a third- or higher-order system. Apparently, G(z) can also be a fractional-order system. Considering a general second-order discrete system:

(38) $$G\left(z\right)=\frac{z+b}{\left(z-1)(z-a\right)},$$

the corresponding iterative algorithm is Equation (25). As mentioned earlier, when setting b=−a/(1+a), one can derive the NAGD. When b=0, one can derive the momentum GD. The iterative algorithm can be viewed as a state-space realization of the corresponding system. Thus, it may have many different realizations (all are equivalent). Since two parameters *a* and *b* are introduced for a general second-order algorithm design, we used the integral squared error (ISE) as the criterion to optimize the parameters. This is because for different target functions f(x), the Lipschitz continuous gradient constant is different. Thus, the loop forward gain is defined as ρ:=μA.

**Figure 13:**
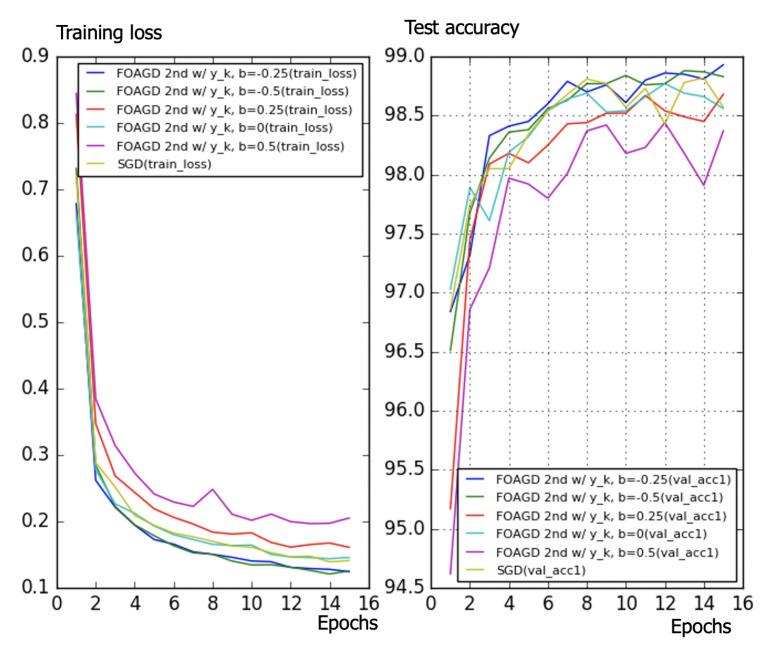
Training loss (**left**); test accuracy (**right**). It is obvious that for different zeros and poles, the performance of the algorithms is different. One finds that both the b=−0.25 and b=−0.5 cases perform better than does the stochastic gradient descent (SGD) momentum. Additionally, both b=0.25 and b=0.5 perform worse. It is also shown that an additional zero can improve the performance, if adjusted carefully.

According to the experimental results (Table 3), interestingly, it is found that the optimal *a* and *b* satisfy b=−a/(1+a), which is the same design as NAGD. Other criteria such as the IAE and ITAE were used to find other optimal parameters, but the results are the same as for the ISE. Differently from for NAGD, the parameters were determined by search optimization rather than by mathematical design, which can be extended to more general cases. The algorithms were then tested using the MNIST dataset (Figure 13). It is obvious that for different zeros and poles, the performance of the algorithms is different. One finds that both the b=−0.25 and b=−0.5 cases perform better than does the SGD momentum. Additionally, both b=0.25 and b=0.5 perform worse. It is also shown that an additional zero can improve the performance, if adjusted properly. It is interesting to observe that both the method and the Nesterov method give an optimal choice of the zero, which is closely related to the pole (b=−a/(1+a)).

Now, let us consider a general third-order discrete system:

(39) $$G\left(z\right)=\frac{z^{2}+cz+d}{\left(z-1\right)\left(z^{2}+az+b\right)}.$$

Set b=d=0; it will reduce to the second-order algorithm discussed above. Compared with the second-order case, the poles can now be complex numbers. More generally, a higher-order system can contain more internal models. If all the poles are real, then:

(40) $$G\left(z\right)=\frac{1}{\left(z-1\right)}\frac{\left(z-c\right)}{\left(z-a\right)}\frac{\left(z-d\right)}{\left(z-b\right)},$$

whose corresponding iterative optimization algorithm is

(41) $$\left\{\begin{array}{c} y_{k+1}=y_{k}-\mu \nabla f\left(x_{k}\right),\\ z_{k+1}=az_{k}+y_{k+1}-cy_{k},\\ x_{k+1}=bx_{k}+z_{k+1}-dz_{k}. \end{array}\right.$$

After some experiments (Table 4), it was found that since the ISE was used for tracking a step signal (it is quite simple), the optimal poles and zeros are the same as for the second-order case with a pole-zero cancellation. This is an interesting discovery. In this optimization result, all the poles and zeros are real, and the resulting performance is not very good, as expected. Compare this with the second-order case; the only difference is that in the latter, complex poles can possibly appear. Thus, the question arises: “how do complex poles play a role in the design?” The answer is obvious: by fractional calculus!

Inspired by M. Jordan’s idea in the frequency domain, a continuous time fractional-order system was designed:

(42) $$G\left(s\right)=\frac{1}{s(s^{\alpha }+\beta )},$$

where α∈(0,2), β∈(0,20] at first. It was then found that the optimal parameters were obtained by searching using the ISE criterion (Table 5).

Equation (42) encapsulates the continuous-time design, and one can use the numerical inverse Laplace transform (NILP) [161] and Matlab command **stmcb( )** [162] to derive its discrete form. After the complex poles are included, one can have:

(43) $$G\left(z\right)=\frac{\left(z+c\right)}{\left(z-1\right)}\left(\frac{1}{z-a+jb}+\frac{1}{z-a-jb}\right)$$

whose corresponding iterative algorithm is:

(44) $$\left\{\begin{array}{c} y_{k+1}=ay_{k}-bz_{k}-\mu \nabla f\left(x_{k}\right),\\ z_{k+1}=az_{k}+by_{k},\\ x_{k+1}=x_{k}+y_{k+1}+cy_{k}. \end{array}\right.$$

**Figure 14:**
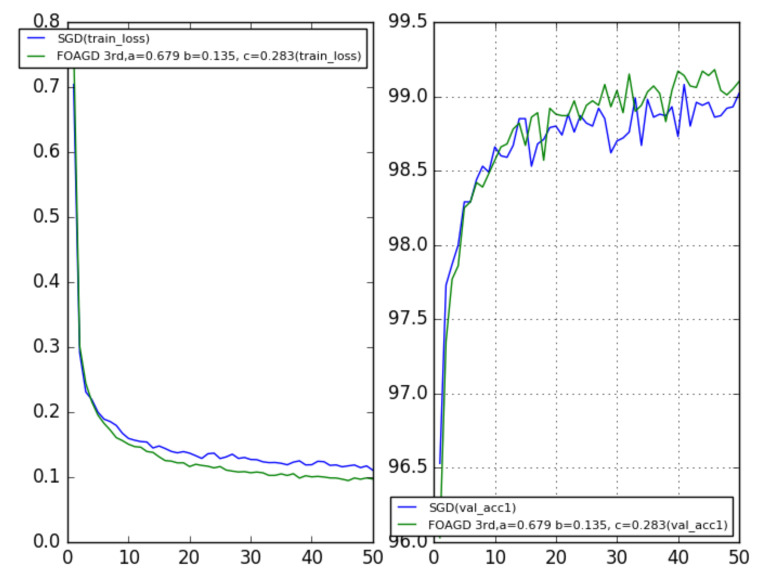
Training loss (**left**); test accuracy (**right**).

Then, the algorithms were tested again using the MNIST dataset, and the results were compared with the SGD’s. For the fractional order, ρ=0.9 was used, a=0.6786, b=0.1354, and different values for zero *c* were used. When c=0, the result was similar to that for the second-order SGD. When *c* was not equal to 0, the result was similar to that for the second-order NAGD. For the SGD, α was set to be 0.9, and the learning rate was 0.1 (Figure 14). Both c=0 and c=0.283 perform better than the SGD momentum; generally, with appropriate values of *c*, better performance can be achieved than in the second-order cases. The simulation results demonstrate that fractional calculus (complex poles) can potentially improve the performance, which is closely related to the learning rate.

In general, M. Jordan asked the question: “is there an optimal way to optimize?”. Our answer is a resounding yes, by limiting dynamics analysis and discretization and SGD with other randomness, such as Langevin motion. Herein, the question posed was: “is there a more optimal way to optimize?”. Again, the answer is yes, but it requires the fractional calculus to be used to optimize the randomness in SGD, random search and the IMP. There is more potential for further investigations along this line of ideas.

## 4. A Case Study of Machine Learning with Fractional Calculus: A Stochastic Configuration Network with Heavytailedness

### 4.1. Stochastic Configuration Network (SCN)

The SCN model is generated incrementally by using stochastic configuration (SC) algorithms [163]. Compared with the existing randomized learning algorithms for single-layer feed-forward neural networks (SLFNNs) [164], the SCN can randomly assign the input weights (w) and biases (b) of the hidden nodes in a supervisory mechanism, which is selecting random parameters with an inequality constraint and assigning the scope of the random parameters adaptively. It can ensure that the built randomized learner models have a universal approximation property. Then, the output weights are analytically evaluated in either a constructive or selective manner [163]. In contrast with the known randomized learning algorithms, such as the randomized radial basis function (RRBF) networks [165] and the random vector functional link (RVFL) [166], the SCN can provide good generalization performance at a faster speed. Concretely, there are three types of SCN algorithms, which are labeled for convenience as SC-I, SC-II and SC-III.

The SC-I algorithm uses a constructive scheme to evaluate the output weights only for the newly added hidden node [167]. All of the previously obtained output weights are kept the same. The SC-II algorithm recalculates part of the current output weights by analyzing a local-least-squares problem with a user-defined shifting window size. The SC-III algorithm finds all the output weights together by solving a global-least-squares problem. The SCN has better performance than other randomized neural networks in terms of fast learning, the scope of the random parameters, and the required human intervention. Therefore, it has already been used in many data-processing projects, such as [134,168,169].

### 4.2. SCN with Heavy-Tailed PDFs

For the original SCN algorithms, weights and biases are randomly generated using a uniform PDF. Randomness plays a significant role in both exploration and exploitation. A good neural network architecture with randomly assigned weights can easily outperform a more deficient architecture with finely tuned weights [170]. Therefore, it is critical to discuss the optimal randomness for the weights and biases in SCN algorithms. Heavy-tailed PDFs have shown optimal randomness for finding targets [171,172], which plays a significant role in exploration and exploitation [148]. Therefore, herein, heavy-tailed PDFs were used to randomly update the weights and biases in the hidden layers to determine if the SCN models display improved performance. Some of the key parameters of the SCN models are listed in Table 6. For example, the maximum times of random configuration T${}_{max}$ are set as 200. The scale factor lambda in the activation function, which directly determines the range for the random parameters, was examined by using different settings (0.5–200). The tolerance was set as 0.05. Most of the parameters for the SCN with heavy-tailed PDFs were kept the same with the original SCN algorithms for comparison purposes. For more details, please refer to [163] and Appendix A.

### 4.3. A Regression Model and Parameter Tuning

The dataset of the regression model was generated by a real-valued function [173]:

(45) $$f\left(x\right)=0.2e^{{-(10x-4)}^{2}}+0.5e^{{-(80x-40)}^{2}}+0.3e^{{-(80x-20)}^{2}},$$

where x ∈ [0, 1]. There were 1000 points randomly generated from the uniform distribution on the unit interval [0, 1] in the training dataset. The test set had 300 points generated from a regularly spaced grid on [0, 1]. The input and output attributes were normalized into [0, 1], and all the results reported in this research represent averages over 1000 independent trials. The settings of the parameters were similar to for the SCN in [163].

Heavy-tailed PDF algorithms have user-defined parameters, for example, the power-law index for SCN-Lévy, and location and scale parameters for SCN-Cauchy and SCN-Weibull, respectively. Thus, to illustrate the effect of parameters on the optimization results and to offer reference values for the proposed SCN algorithms, parameter analysis was conducted, and corresponding experiments were performed. Based on the experimental results, for the SCN-Lévy algorithm, the most optimal power-law index is 1.1 for achieving the minimum number of hidden nodes. For the SCN-Weibull algorithm, the optimal location parameter α and scale parameter β for the minimum number of hidden nodes are 1.9 and 0.2, respectively. For the SCN-Cauchy algorithm, the optimal location parameter α and scale parameter β for the minimum number of hidden nodes are 0.9 and 0.1, respectively.

#### Performance Comparison among SCNs with Heavy-Tailed PDFs

**Figure 15:**
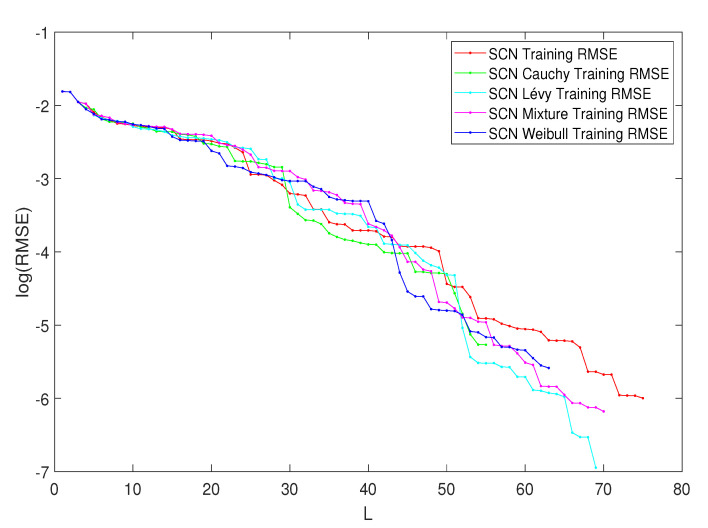
Performance of SCN, SCN-Lévy, SCN-Weibull, SCN-Cauchy and SCN-Mixture. The parameter L is the hidden node number.

In Table 7, the performance of SCN, SCN-Lévy, SCN-Cauchy, SCN-Weibull and SCN-Mixture are shown, in which mean values are reported based on 1000 independent trials. Wang et al. [163] used time cost to evaluate the SCN algorithms’ performance. In the present study, the authors used the mean hidden node numbers to evaluate the performance. The number of hidden nodes is associated with modeling accuracy. Therefore, herein, the analysis determined if an SCN with heavy-tailed PDFs used fewer hidden nodes to generate high performance, which would make the NNs less complex. According to the numerical results, the SCN-Cauchy used the lowest number of mean hidden nodes, 59, with an root mean squared error (RMSE) of 0.0057. The SCN-Weibull had a mean number of 63 hidden nodes, with an RMSE of 0.0037. The SCN-Mixture had a mean number of 70 hidden nodes, with an RMSE of 0.0020. The mean number of hidden nodes for SCN-Lévy was also 70. The original SCN model had a mean number of 75 hidden nodes. A more detailed training process is shown in Figure 15. With fewer hidden node numbers, the SCN models with heavy-tailed PDFs can be faster than the original SCN model. The neural network structure is also less complicated than the SCN. Our numerical results for the regression task demonstrate remarkable improvements in modeling performance compared with the current SCN model results.

### 4.4. MNIST Handwritten Digit Classification

**Figure 16:**
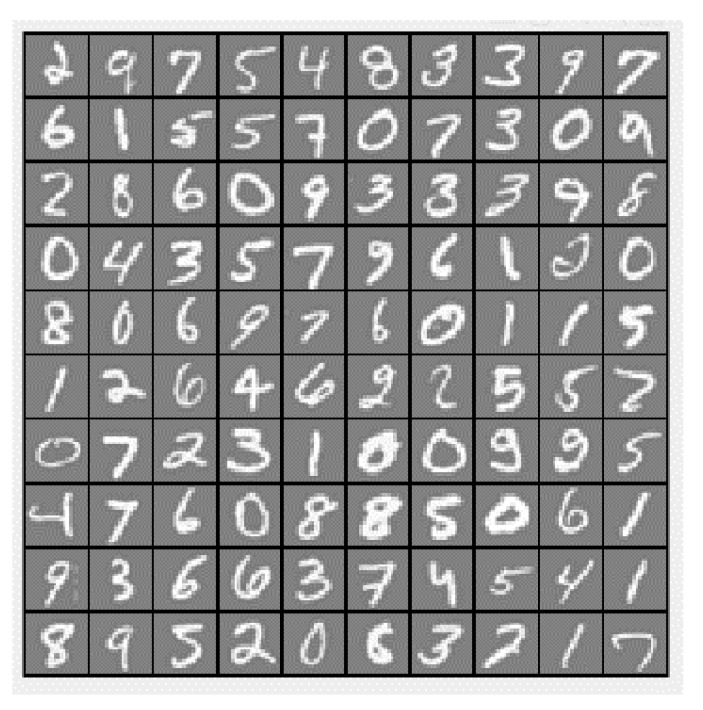
The handwritten digit dataset example.

The handwritten digit dataset contains 4000 training examples and 1000 testing examples, a subset of the MNIST handwritten digit dataset. Each image is a 20-by-20-pixel grayscale image of the digit (Figure 16). Each pixel is represented by a number indicating the grayscale intensity at that location. The 20-by-20 grid of pixels is “unrolled” into a 400-dimensional vector.

Similar to the parameter tuning for the regression model, parameter analysis was conducted to illustrate the impact of parameters on the optimization results and to offer reference values for the MNIST handwritten digit classification SCN algorithms. Corresponding experiments were performed. According to the experimental results, for the SCN-Lévy algorithm, the most optimal power law index is 1.6 for achieving the best RMSE performance. For the SCN-Cauchy algorithm, the optimal location parameter α and scale parameter β for the lowest RMSE are 0.2 and 0.3, respectively.

#### Performance Comparison among SCNs on MNIST

**Figure 17:**
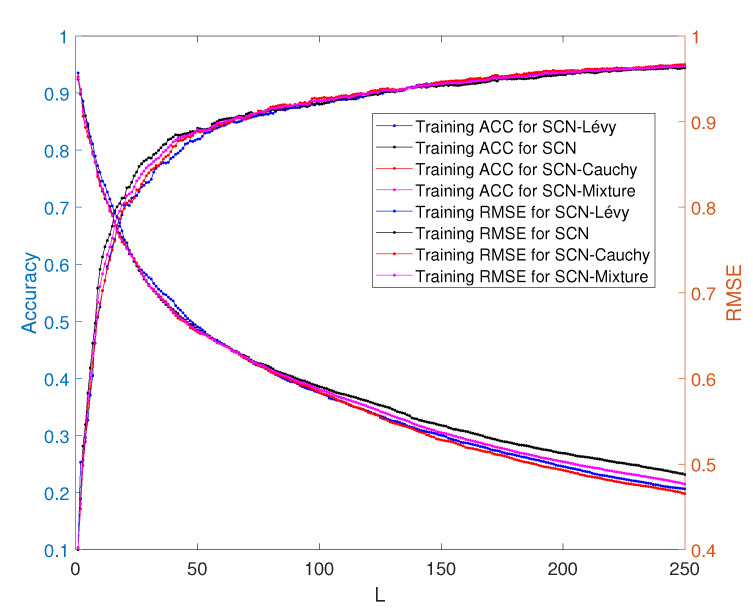
Classification performance of SCNs.

The performance of the SCN, SCN-Lévy, SCN-Cauchy and SCN-Mixture are shown in Table 8. Based on the experimental results, the SCN-Cauchy, SCN-Lévy and SCN-Mixture have better performance in training and test accuracy, compared with the original SCN model. A detailed training process is shown in Figure 17. Within around 100 hidden nodes, the SCN models with heavy-tailed PDFs perform similarly to the original SCN model. When the number of the hidden nodes is greater than 100, the SCN models with heavy-tailed PDFs have lower RMSEs. Since more parameters for weights and biases are initialized in heavy-tailed PDFs, this may cause an SCN with heavy-tailed PDFs to converge to the optimal values at a faster speed. The experimental results for the MNIST handwritten classification problem demonstrate improvements in modeling performance. They also show that SCN models with heavy-tailed PDFs have a better search ability for achieving lower RMSEs.

## 5. Take-Home Messages and Looking into the Future: Fractional Calculus Is Physics Informed

Big data and machine learning (ML) are two of the hottest topics of applied scientific research, and they are closely related to one another. To better understand them, in this article, we advocate fractional calculus (FC), as well as fractional-order thinking (FOT), for big data and ML analysis and applications. In Section 2, we discussed the relationships between big data, variability and FC, as well as why fractional-order data analytics (FODA) should be used and what it is. The topics included the Hurst parameter, fractional Gaussian noise (fGn), fractional Brownian motion (fBm), the fractional autoregressive integrated moving average (FARIMA), the formalism of continuous time random walk (CTRW), unmanned aerial vehicles (UAVs) and precision agriculture (PA).

In Section 3, how to learn efficiently (optimally) for ML algorithms is discussed. The key to developing an efficient learning process is the method of optimization. Thus, it is important to design an efficient optimization method. The derivative-free methods, as well as the gradient-based methods, such as the Nesterov accelerated gradient descent (NAGD), are discussed. Furthermore, it is shown to be possible, following the internal model principle (IMP), to design and analyze the ML algorithms in the S or Z transform domain in Section 3.3. FC is used in optimal randomness in the methods of stochastic gradient descent (SGD) and random search. Nonlocal models have commonly been used to describe physical systems and/or processes that cannot be accurately described by classical approaches [174]. For example, fractional nonlocal Maxwell’s equations and the corresponding fractional wave equations were applied in [175] for fractional vector calculus [176]. The nonlocal differential operators [177], including nonlocal analogs of the gradient/Hessian, are the key of these nonlocal models, which could lead to very interesting research with FC in the near future.

**Figure 18:**
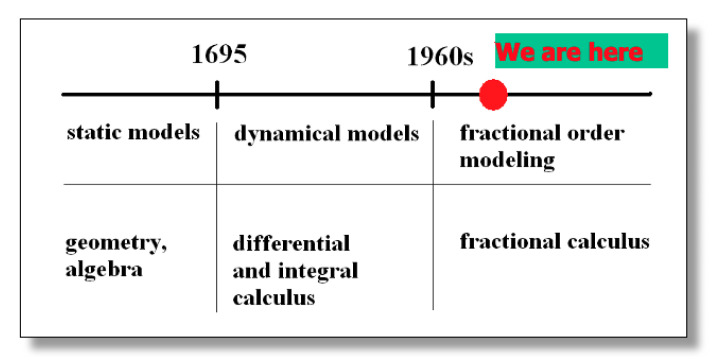
Timeline of FC (courtesy of Professor Igor Podlubny).

Fractional dynamics is a response to the need for a more advanced characterization of our complex world to capture structure at very small or very large scales that had previously been smoothed over. If one wishes to obtain results that are better than the best possible using integer-order calculus-based methods, or are “more optimal”, we advocate applying FOT and going fractional! In this era of big data, decision and control need FC, such as fractional-order signals, systems and controls. The future of ML should be physics-informed, scientific (cause–effect embedded or cause–effect discovery) and involving the use of FC, where the modeling is closer to nature. Laozi (unknown, around the 6th century to 4th century BC), the ancient Chinese philosopher, is said to have written a short book DaoDeJing(TaoTeChing), in which he observed: “The Tao that can be told is not the eternal Tao” [178]. People over thousands of years have shared different understandings of the meaning of the Tao. Our best understanding of the Tao is nature, whose rules of complexity can be explained in a non-normal way. Fractional dynamics, FC and heavytailedness may well be that non-normal way (Figure 18), at least for the not-too-distant future.

## Figures and Tables

**Table 1:** The 10 Vs of big data.

Characteristics	Description
1. Volume	Best known characteristic of big data; more than 90 percent of the whole data were created in the past couple of years.
2. Velocity	The speed at which data are being generated.
3. Variety	Processing structured, unstructured and semistructured data.
**4. Variability**	Inconsistent speed of data loading, multitude of data dimensions, and number of inconsistencies.
5. Veracity	Confidence or trust in the data.
6. Validity	Refers to how accurate and correct the data are.
7. Vulnerability	Security concerns, data breaches.
8. Volatility	Design policy for data currency, availability, and rapid retrieval of information when required.
9. Visualization	Develop new tools considering the complex relationships between the above properties.
10. Value	The most important of the 10 Vs; substantial value must be found.

**Table 2:** Variability in multiple research topics.

Topics	Description
1. Climate variability	Changes in the components of the climate system and their interactions.
2. Genetic variability	Measurements of the tendencies of individual genotypes between regions.
3. Heart rate variability	Physiological phenomenon where the time interval between heart beats varies.
4. Human variability	Measurements of the characteristics, physical or mental, of human beings.
5. Spatial variability	Measurements at different spatial points exhibit different values.
6. Statistical variability	A measure of dispersion in statistics.

**Table 3:** General second-order algorithm design. The parameter ρ is the loop forward gain; see text for more details.

ρ	0.4	0.8	1.2	1.6	2.0	2.4
a	−0.6	−0.2	0.2	0.6	1	1.4
b	1.5	0.25	−0.1667	−0.3750	−0.5	−0.5833

**Table 4:** General third-order algorithm design, with parameters defined by Equation (41).

ρ	0.4	0.8	1.2	1.6	2.0	2.4
a	0.6439	0.5247	−0.4097	−0.5955	−1.0364	−1.4629
b	0.0263	0.0649	0.0419	−0.0398	0.0364	0.0880
c	1.5439	0.5747	−0.3763	−0.3705	−0.5364	−0.6462
d	0.0658	0.0812	0.0350	−0.0408	0.0182	0.0367

**Table 5:** The continuous time fractional-order system.

ρ	0.3	0.5	0.7	0.9
α	1.8494	1.6899	1.5319	1.2284
β	20	20	20	20

**Table 6:** Stochastic configuration networks (SCNs) with key parameters.

Properties	Values
Name:	“Stochastic Configuration Networks”
Version:	“1.0 beta”
L:	hidden node number
W:	input weight matrix
b:	hidden layer bias vector
Beta:	output weight vector
r:	regularization parameter
tol:	tolerance
Lambda:	random weight range
L${}_{max}$:	maximum number of hidden neurons
T${}_{max}$:	maximum times of random configurations
nB:	number of node being added in one loop

**Table 7:** Performance comparison of SCN models for regression problem.

Models	Mean Hidden Node Number	RMSE
SCN	75 ± 5	0.0025
SCN-Lévy	70 ± 6	0.0010
SCN-Cauchy	59 ± 3	0.0057
SCN-Weibull	63 ± 4	0.0037
SCN-Mixture	70 ± 5	0.0020

**Table 8:** Performance comparison of SCNs.

Models	Training Accuracy	Test Accuracy
SCN	94.0 ± 1.9%	91.2 ± 6.2%
SCN-Lévy	94.9 ± 0.8%	91.7 ± 4.5%
SCN-Cauchy	95.4 ± 1.3%	92.4 ± 5.5%
SCN-Mixture	94.7 ± 1.1%	91.5 ± 5.3%

## Data Availability

Not applicable.

## Abbreviations

The following abbreviations are used in this manuscript:

ACF	Auto-Correlation Function
AI	Artificial Intelligence
ARMA	Autoregression and Moving Average
CLT	Classical Central Limit Theorem
CS	Cuckoo Search
CTRW	Continuous Time Random Work
EOM	Equation of Motion
fBm	Fractional Brownian Motion
fGn	Fractional Gaussian Noise
FARIMA	Fractional Autoregressive Integrated Moving Average
FC	Fractional Calculus
FIGARCH	Fractional Integral Generalized Autoregressive Conditional Heteroscedasticity
FLOM	Fractional Lower-Order Moments
FOCV	Fractional-Order Calculus of Variation
FODA	Fractional-Order Data Analytics
FOEL	Fractional-Order Euler–Lagrange
FOT	Fractional-Order Thinking
GARMA	Gegenbauer Autoregressive Moving Average
GD	Gradient Descent
GDM	Gradient Descent Momentum
GEV	Generalized Extreme Value
IMP	Internal Model Principle
IPL	Inverse Power Law
ISE	Integral Squared Error
LGD	Long Range Dependence
LTI	Linear Time Invariant
MAD	Modeling, Analysis and Design
ML	Machine Learning
MLL	Mittag–Leffler Law
MNIST	Modified National Institute of Standards and Technology Database
NAGD	Nesterov Accelerated Gradient Descent
NDVI	Normalized Difference Vegetation Index
NILT	Numerical Inverse Laplace Transform
NN	Neural Networks
PA	Precision Agriculture
PDF	Probability Density Function
PID	Proportional, Integral, Derivative
PSO	Particle Swarm Optimization
RBF	Randomized Radial Basis Function (RBF) Networks
RGB	Red, Green, Blue
RMSE	Root Mean Squared Error
RVFL	Random Vector Functional Link
RW-FNN	Feed-Forward Networks with Random Weights
SCN	Stochastic Configuration Network
SGD	Stochastic Gradient Descent
SLFNNs	Single-Layer Feed-Forward Neural Networks
UAVs	Unmanned Aerial Vehicles
USDA	United States Department of Agriculture
wGn	White Gaussian Noise

## Appendix A. SCN Codes

The Matlab and Python codes can be found at https://github.com/niuhaoyu16/StochasticConfigurationNetwork (accessed on 2 February 2021).
